# Multi-Source Remote Sensing and Ensemble Learning for Habitat Suitability Mapping of the Common Leopard (*Panthera pardus*) in Azad Jammu and Kashmir, Pakistan

**DOI:** 10.3390/s26103088

**Published:** 2026-05-13

**Authors:** Zeenat Dildar, Wenjiang Huang, Raza Ahmed, Zeeshan Khalid

**Affiliations:** 1 State Key Laboratory of Remote Sensing Science, Aerospace Information Research Institute, Chinese Academy of Sciences, Beijing 100094, China; zeenatdildar123@mails.ucas.ac.cn; 2 University of Chinese Academy of Sciences, Beijing 100049, China; 3 Sino-UK Pest and Disease Forecasting & Management Joint Laboratory, Beijing 100094, China; 4 WWF-Pakistan, Ferozepur Road, P.O. Box 5180, Lahore 54600, Pakistan; chz39@hotmail.com

**Keywords:** remote sensing, habitat suitability modeling, ensemble machine learning, spatial ecological modeling, Earth observation data, habitat mapping, geospatial analysis, common leopard (*Panthera pardus*), mountain ecosystems, biodiversity conservation

## Abstract

**Highlights:**

**What are the main findings?**

- Ensemble habitat suitability modeling, combining multiple remote sensing data layers, identified approximately 30% of the study area as highly suitable habitat for the common leopard, predominantly in the northern and central regions.
- Key environmental drivers identified for leopard habitat suitability included land cover, elevation, fractional vegetation cover, slope, and temperature seasonality, all derived from remote sensing data.

**What are the implications of the main findings?**

- Identifying high-suitability habitat areas provides a scientific foundation for prioritizing conservation efforts, maintaining habitat connectivity, and informing land-use management in Azad Jammu and Kashmir.
- The dominance of vegetation, topography, and climatic variables in habitat suitability highlights the critical role of remote sensing-derived environmental variables in effective wildlife monitoring and assessing ecological responses to environmental changes.

**Abstract:**

Remote sensing technologies provide valuable geospatial data for analyzing environmental conditions and for supporting spatial ecological modeling across large, heterogeneous landscapes. In this study, multi-source remote sensing–derived environmental variables were integrated with ensemble machine learning techniques to model the habitat suitability of the common leopard (*Panthera pardus*) in Azad Jammu and Kashmir (AJ&K), Pakistan. Environmental predictors derived from satellite observations included land cover, vegetation condition, terrain attributes, and climate-related indicators. To ensure model reliability, multicollinearity among predictors was evaluated, and spatial clustering patterns of leopard occurrence records were examined using global spatial autocorrelation analysis. Two complementary machine learning algorithms, Maximum Entropy (MaxEnt) and Random Forest (RF), were implemented and integrated through a weighted ensemble approach to improve predictive accuracy and robustness. The ensemble model achieved high predictive performance with an area under the curve (AUC) value of 0.942, outperforming individual algorithms. The resulting habitat suitability map indicates that approximately 30% of the study region is highly suitable habitat, primarily in the northern and central districts, including Muzaffarabad, Neelum, Hattian, Poonch, and Sudhnutti. Variable importance analysis identified remotely sensed land cover, elevation, vegetation cover, slope, and temperature seasonality as the dominant predictors of habitat suitability, whereas anthropogenic indicators such as proximity to roads and population density had secondary effects in fragmented areas. The results demonstrate the potential of integrating remote sensing data and ensemble machine learning for spatial habitat modeling and wildlife conservation planning in mountainous ecosystems.

## 1. Introduction

Habitat availability and quality are fundamental determinants of wildlife persistence, governing access to essential resources such as food, shelter, and reproductive sites [1,2]. For large carnivores, whose ecological roles and spatial requirements are substantial, even modest habitat alterations can cause pronounced population declines [3,4,5]. Across much of their range, natural habitats are increasingly transformed by anthropogenic pressures, including land-use change, deforestation, infrastructure expansion, and direct persecution [6,7,8]. These pressures have led to widespread habitat loss and fragmentation, undermining the long-term viability of many apex predators [9,10].

The common leopard (*Panthera pardus*) is one of the most widely distributed large felids, exhibiting remarkable ecological flexibility across diverse landscapes [11,12,13]. Despite this adaptability, leopard populations are declining in many regions due to escalating human pressures and environmental change [11,14]. Climate-driven shifts in temperature and precipitation are expected to alter further vegetation patterns, prey distribution, and forest structure, potentially reducing the extent of suitable leopard habitat [15,16,17,18]. As habitats become increasingly fragmented, prey availability may decline, and spatial connectivity between suitable areas may be disrupted, forcing leopards to move closer to human-dominated landscapes [19,20]. These processes often intensify human–leopard interactions, leading to livestock depredation, retaliatory killings, and reduced tolerance for the species [21].

Human–carnivore conflict has emerged as a major conservation challenge worldwide, particularly in regions where expanding human populations overlap with large predator habitats [22,23,24]. Prolonged conflicts threaten carnivore survival and erode public support for conservation initiatives. Studies across Asia and Africa indicate that large felids, including leopards, are frequently involved in conflicts at rates disproportionate to their abundance [25,26,27]. These interactions underscore the urgent need to identify and protect suitable habitats that can sustain viable populations while minimizing conflict risk [25,28].

In Pakistan, the common leopard inhabits fragmented, heterogeneous landscapes ranging from arid highlands to moist temperate forests [29,30]. Azad Jammu and Kashmir (AJ and K) is a critical stronghold for the species, supported by rugged topography, forest cover, and lower human density than surrounding regions [30,31]. However, expanding infrastructure, forest degradation, prey depletion, and human encroachment are progressively degrading habitat quality in the area [32,33,34]. Nationally, the conservation status of the common leopard is more severe than its global classification, underscoring the importance of region-specific assessments to guide effective management and conservation strategies [35,36].

Species Distribution Models (SDMs) have become indispensable tools for assessing habitat suitability and predicting species distributions under current and future environmental conditions [37,38]. By integrating species occurrence data with environmental variables, SDMs provide spatially explicit insights into habitat preferences and potential distribution patterns [39,40]. Among the various modeling techniques, Maximum Entropy (MaxEnt) has been widely used with presence-only data, particularly for elusive and rare species [41]. In contrast, machine-learning algorithms such as Random Forest (RF) are well suited to capturing complex, nonlinear relationships between species occurrence and environmental predictors, especially in ecologically heterogeneous landscapes [42].

Recent advances in ecological modeling emphasize the value of ensemble approaches that combine multiple algorithms to improve predictive accuracy and reduce uncertainty inherent in individual models [43,44]. Ensemble modeling leverages the complementary strengths of different techniques, often yielding more robust and reliable habitat suitability predictions [43,45]. Despite the growing adoption of ensemble SDMs worldwide, their application to assessing leopard habitat suitability in Pakistan—particularly in AJ and K—remains limited.

In this study, we apply an ensemble modeling framework, complemented by spatial autocorrelation analysis, to assess the habitat suitability of the common leopard across Azad Jammu and Kashmir. Using verified leopard occurrence records and multi-source environmental data, this research aims to: (1) map the spatial distribution of suitable leopard habitats across the region; (2) identify and quantify the relative importance of key environmental and anthropogenic factors influencing leopard distribution; (3) evaluate the predictive performance of individual and ensemble model; and (4) delineate priority areas for conservation and management interventions. The findings of this study are expected to provide a robust scientific basis for proactive conservation planning, habitat protection, and human–wildlife conflict mitigation strategies for the common leopard in this ecologically important region.

## 2. Materials and Methods

**Figure 1:**
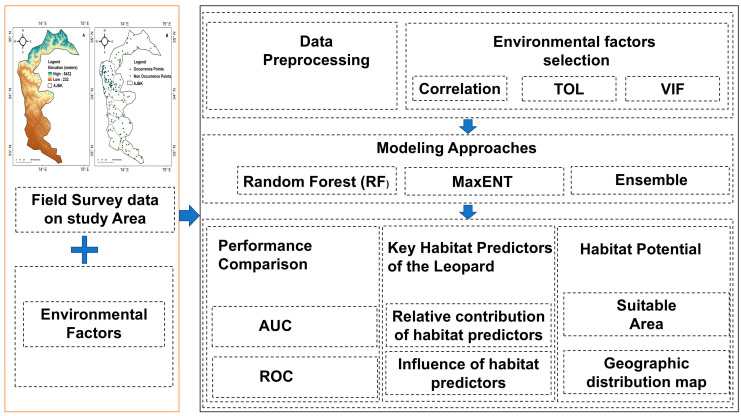
Analysis process. (**A**) Elevation of the study area; (**B**) Leopard points in the study area. First orange box shows Field survey data and environmental factors used for habitat suitability analysis in the study area. Second black box is a modeling framework showing data preprocessing, environmental factor selection using correlation, tolerance (TOL), and variance inflation factor (VIF), followed by habitat suitability modeling using Random Forest (RF), MaxENT, and Ensemble approaches, including performance evaluation (AUC and ROC), identification of key habitat predictors, and habitat potential mapping.

The analytical approach for this study involved several steps to identify the key environmental factors influencing the spatial distribution and habitat suitability of the common leopard (*P. pardus*) in Azad Jammu and Kashmir (AJ and K) (Figure 1). The process started with the careful selection of ecologically relevant environmental variables sourced from various datasets, including climate, topography, vegetation, and human activities. These variables were chosen based on their known relationships to leopard habitat needs, and additional field observations within the known range supported this selection. Once the relevant variables were selected, all spatial datasets were standardized to a consistent 1 km^2^ resolution using a Python-based geo-processing script in ArcGIS 10.8. This standardization ensured compatibility in spatial scale and coordinate systems, enabling accurate comparisons across variables. Environmental variables were resampled using nearest-neighbor interpolation to ensure consistency across datasets. To explore relationships among the selected predictors, Pearson and Spearman’s rank correlation were used. This non-parametric method was chosen because it can detect monotonic relationships, which are common in ecological data. Variables with strong correlations (|r| > 0.7) were considered redundant and were removed to prevent multicollinearity from affecting the models. Next, the Variance Inflation Factor (VIF) and Tolerance (TOL) tests were applied to further assess multicollinearity. Variables with VIFs > 5 (or TOL < 0.2) were iteratively removed to ensure that the remaining predictors were independent and meaningful. For habitat suitability modeling, a weighted ensemble approach was used to combine MaxEnt and Random Forest (RF) models. The final habitat suitability map was created by merging the results of both models through a weighted ensemble method. In this method, the predictions from each model were weighted by their Area Under the Curve (AUC) values from Receiver Operating Characteristic (ROC) analysis, ensuring that the more accurate model had greater influence on the final output. This approach leverages the strengths of both models to produce a more robust and reliable habitat suitability map for the common leopard. The performance of the individual models (MaxEnt and RF) and the ensemble model was evaluated using ROC curves and the AUC metric. AUC values offered a comprehensive measure of model accuracy, with higher values indicating better predictive ability. Additionally, a variable importance analysis was conducted to assess the relative contribution of each environmental factor to the final model predictions. This analysis helped identify the most influential ecological drivers affecting leopard habitat suitability in the region.

### 2.1. Study Area

**Figure 2:**
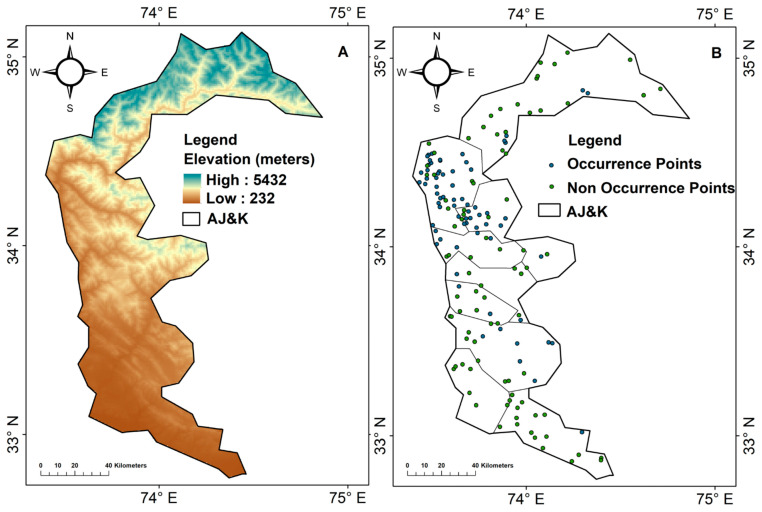
Location map of the study area. (**A**) Elevation of the study area; (**B**) Leopard points in the study area.

Azad Jammu and Kashmir (AJ&K) in northern Pakistan (32–36° N, 73–75° E) (Figure 2) spans approximately 13,300 km^2^ of predominantly mountainous terrain in the western Himalaya, recognized as a regional biodiversity hotspot [46,47]. Elevations range from low hills (232 m) to high peaks (5432 m), generating sharp climatic and vegetation gradients from subtropical woodlands to moist temperate and alpine forests [48].

Administratively, AJ&K comprises ten districts, with comparatively low-relief, densely settled landscapes in the south (Bhimber, Mirpur, Kotli) and steep, forested terrain in the north (Muzaffarabad, Hattian, Neelum, Bagh, Haveli, Poonch, Sudhnoti) [30]. These north–south contrasts in topography and land cover underpin strong spatial gradients in human disturbance, fragmentation, and potential leopard habitat, consistent with broader patterns in northern Pakistan [36].

The regional climate ranges from dry subtropical in the southern districts to moist temperate and cold conditions at higher elevations, with annual precipitation typically between ~1000 and 2000 mm and a strong monsoonal component [49,50]. Three major river systems—Jhelum, Neelum, and Poonch—structure valley agriculture and settlement, creating a mosaic of mixed forest, agro-forest, rangeland, and built-up areas that is well captured by contemporary optical remote sensing and digital elevation model (DEM) products used in species distribution modelling [51,52]. AJ&K also hosts a network of protected areas and game reserves, though their effectiveness and coverage of high-quality carnivore habitat remain limited, similar to patterns reported for leopard landscapes elsewhere in Asia [19,53].

### 2.2. Data Collection and Field Observations

Data for the common leopard (*P. pardus*) were gathered across Azad Jammu and Kashmir (AJ and K) from 2018 to 2023 using an integrative, incident-driven approach. Instead of conducting predefined transect surveys, data were collected based on reports of leopard-related incidents, particularly livestock depredation and retaliatory killings. These reports were sourced from various channels, including local informants, field officers, and official records from the AJ and K Wildlife Department. To ensure data accuracy, each incident was cross-verified against multiple independent sources, including interviews with local herders, community leaders, and wildlife experts, as well as media reports.

Field teams visited the reported locations to conduct focused observations, using Garmin GPSMAP 72H handheld devices to record the precise locations of leopard occurrences with an accuracy of ±7 m. Indirect signs of leopard presence, such as scat, pugmarks, scratch marks, and territorial markings, were identified and documented according to established field protocols [12,54,55]. To further reduce the risk of misidentification, the observations were validated through consultations with local wildlife experts and community members. In addition, structured interviews were conducted with eyewitnesses and livestock owners to gather supplementary data on depredation events, retaliation, and historical leopard sightings [56,57]. In total, 70 verified occurrence points were identified, representing confirmed locations of leopard presence. To complement this, 88 non-occurrence points were selected from areas where no leopard presence was reported. These non-occurrence points were chosen to reflect the geographical context of potential leopard habitats, ensuring they were representative of regions with similar environmental conditions but lacking confirmed leopard presence, thereby enhancing the accuracy of habitat suitability modeling.

Environmental data to support the modeling process included 19 bioclimatic variables (Bio1–Bio19) sourced from WorldClim 2.1 at a 1 km resolution, derived from long-term temperature and precipitation records. Additional specific humidity data were obtained from the Famine Early Warning Systems Network (FEWS NET) Land Data Assimilation System (FLDAS) dataset at an 11,132 km^2^ resolution. Vegetation and land cover data were sourced from MODIS products, including the Normalized Difference Vegetation Index (NDVI) from the MOD13A2.061 product and land cover classification from the MCD12Q1.061 dataset. Topographic data, including elevation, slope, and aspect, were extracted from the GDEM V2 and V3 datasets. Anthropogenic factors, such as distance to roads and population density, were sourced from the Humanitarian Data Exchange (HDX) at 1 km resolution. In contrast, hydrological data, including river distance, were extracted from the HYDROSHEDS database and resampled to 1 km resolution. A detailed description of all 28 environmental variables and their data sources is provided in Table 1. All environmental datasets were standardized to a 1 km resolution to ensure consistency and compatibility across variables, thereby facilitating accurate model predictions.

### 2.3. Multicollinearity Evaluation

A Spearman correlation analysis was performed to evaluate the relationship between all selected variables. Variables with high correlation (|r| > 0.7) were removed to prevent multicollinearity [58]. Additionally, Variance Inflation Factor (VIF) and Tolerance (TOL) were calculated for the remaining variables, and any with VIF > 5 (equivalent to TOL < 0.2) were eliminated [59]. This process ensured that the variables used in the modeling were independent and ecologically meaningful.

### 2.4. Evaluation of Global Spatial Autocorrelation in Common Leopard Occurrence

To quantify the spatial structure of common leopard occurrence across the study area, Global Moran’s I was used to measure spatial autocorrelation. This method assesses whether observed spatial patterns are clustered, dispersed, or randomly distributed by comparing the similarity of values among neighboring spatial units [60].

Occurrence data for the common leopard were converted into a spatial point dataset and analyzed using a spatial weights matrix based on neighborhood proximity. Moran’s I values range from −1 to +1, where values close to +1 indicate strong clustering, values near −1 represent spatial dispersion, and values around zero suggest a random spatial pattern [61,62].

Statistical significance was evaluated using the corresponding z-score and *p*-value, derived through permutation testing [63]. A high positive Moran’s I value accompanied by a significant z-score indicates that similar values (presence locations) are spatially clustered rather than randomly distributed. This approach provides a robust assessment of global spatial dependence and serves as a foundational step for subsequent local spatial analyses and habitat suitability modeling.

### 2.5. Ensemble Modeling Approach

For habitat suitability modeling, we applied an ensemble approach that integrates two robust machine learning algorithms: Maximum Entropy (MaxEnt) and Random Forest (RF). This ensemble framework was chosen to combine the strengths of both algorithms, allowing for more accurate and reliable predictions of leopard habitat suitability.

The MaxEnt model, well-suited for presence-only data, predicts species distributions by maximizing entropy, thus estimating the most probable habitat distribution based on environmental variables. To ensure robustness, we implemented a bootstrapping procedure with 50 iterations, randomly partitioning the data into training (70%) and testing (30%) subsets while maintaining spatial stratification. The default settings were used for most parameters, optimizing performance while ensuring generalizability.

For the RF model, which captures complex nonlinear relationships between ecological variables, we applied a 5-fold cross-validation grid search to optimize key hyperparameters, including the number of trees (n_estimators: 100, 300, 500), maximum depth of trees (max_depth: 10, 20, None), and the minimum number of samples required to split a node (min_samples_split: 2, 5). The final model used 500 trees, based on results showing optimal performance in preliminary tests and previous research indicating stable performance beyond 300–500 trees [64]. To mitigate class imbalance, balanced class weights were also incorporated. Log loss was used as the performance metric during tuning to ensure the model accurately captured habitat variability without overfitting.

We then combined the habitat suitability predictions from MaxEnt and RF using a weighted average, with each model’s prediction weighted by its AUC from Receiver Operating Characteristic (ROC) analysis. This approach capitalizes on the complementary strengths of MaxEnt and RF, combining MaxEnt’s probabilistic estimates with RF’s ability to capture complex, nonlinear patterns.

The final ensemble habitat suitability map (H) was generated using the following equation:

(1) $$H=\frac{\left(H_{\mathrm{MaxEnt}}\times w_{\mathrm{MaxEnt}})+(H_{\mathrm{RF}}\times w_{\mathrm{RF}}\right)}{w_{\mathrm{MaxEnt}}+w_{\mathrm{RF}}}$$

Here $H_{\mathrm{MaxEnt}}$ and $H_{\mathrm{RF}}$ represent the habitat suitability predictions from the MaxEnt and RF models, respectively, $w_{\mathrm{MaxEnt}}$ and $w_{\mathrm{RF}}$ are the performance weights of each model, calculated based on their respective Area Under the Curve (AUC) values from ROC analysis.

This ensemble approach provides a more robust estimate of leopard habitat suitability by leveraging the complementary strengths of both models. The final habitat suitability map was classified into three suitability classes: less suitable, moderately suitable, and most suitable [65,66]. To identify the key predictors influencing habitat suitability, we assessed feature importance using model-derived contribution metrics within the ensemble framework [67]. These contributions were used to quantify the relative influence of each environmental variable on habitat suitability predictions. The resulting feature importance values provide an interpretable measure of the dominant factors shaping species distribution patterns [68].

## 3. Results

### 3.1. Multicollinearity

**Figure 3:**
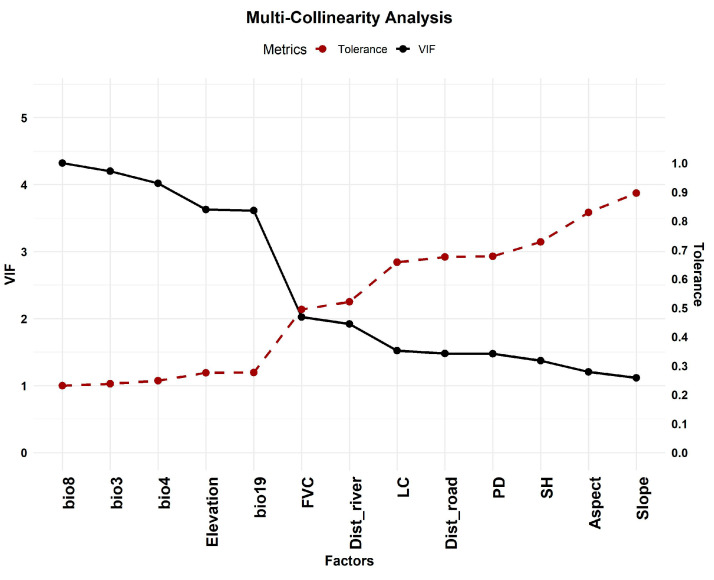
The VIF and TOL graphs of factors.

**Figure 4:**
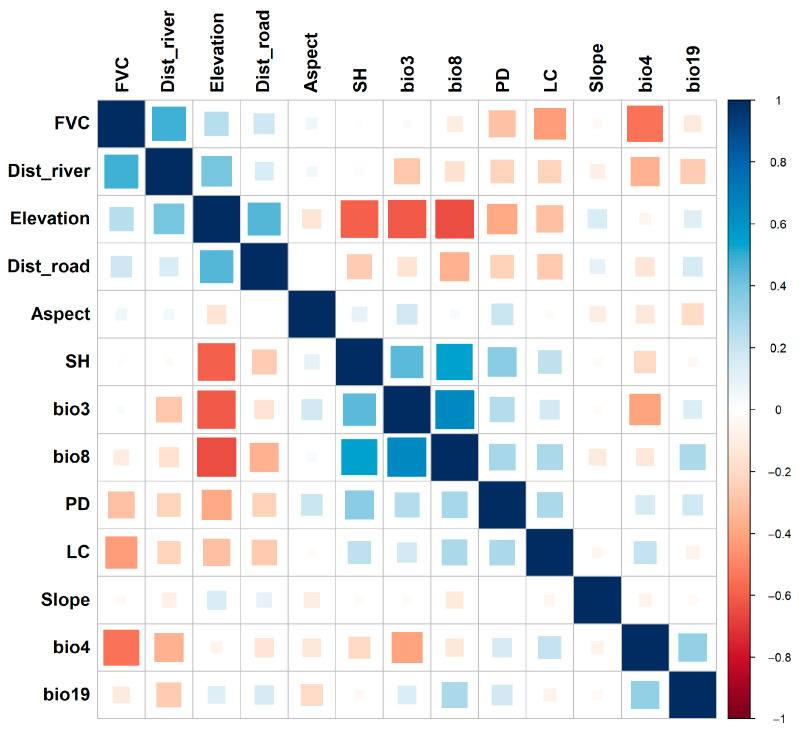
Pearson correlation.

**Figure 5:**
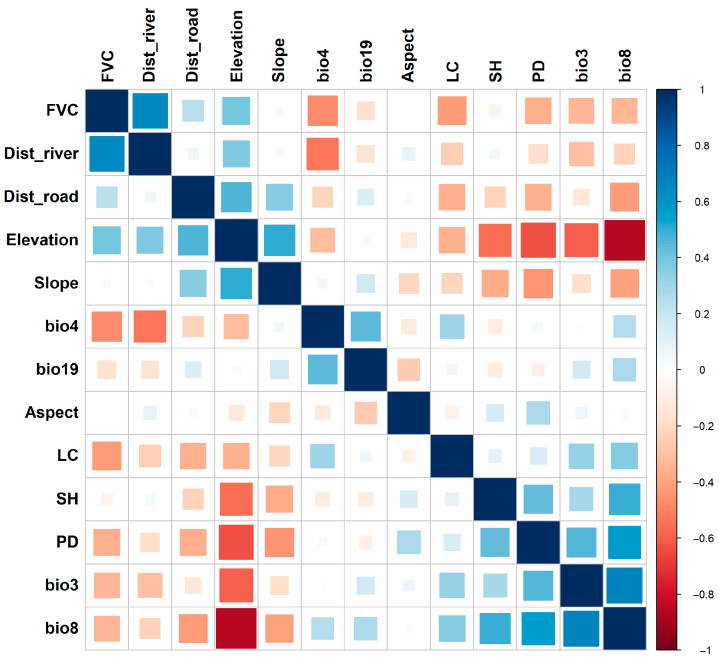
Spearman correlation.

A comprehensive assessment of multicollinearity (Figure 3, Figure 4 and Figure 5) was performed to ensure the stability and interpretability of the environmental predictors used in the MaxEnt, Random Forest (RF), and ensemble habitat suitability models. The analysis used the Variance Inflation Factor (VIF) and Tolerance (TOL) metrics, complemented by Pearson’s and Spearman’s correlation matrices. All results confirmed the absence of significant multicollinearity. The VIF values for all predictors remained well below the 5 threshold, ranging from 1.116 (Slope) to 4.321 (bio8). Likewise, TOL values were above the critical value of 0.1, with a minimum of 0.238 (bio3). Additionally, Pearson’s and Spearman’s correlation coefficients showed no strong linear or monotonic relationships, as none exceeded the |0.7| threshold. Therefore, multicollinearity is not an issue for this dataset. As a result, 13 factors were chosen for analysis: FVC, Dist_river, Elevation, Dist_road, Aspect, SH, bio3, bio8, PD, LC, Slope, bio4, and bio19.

### 3.2. Global Spatial Autocorrelation of Common Leopard Occurrence

The global spatial autocorrelation analysis indicated a significant clustered pattern in the occurrence of the common leopard (*P. pardus*) across the study area (Figure S1). The Moran’s Index value (0.746) reflects strong positive spatial autocorrelation. The corresponding z-score (2.749) exceeds the critical threshold at the 0.01 significance level, and the *p*-value (0.005975) confirms that the observed pattern is unlikely to have occurred by random chance.

These findings demonstrate that common leopard occurrence points are spatially clustered, suggesting a non-random distribution driven by spatially structured environmental or ecological factors that influence leopard presence within the landscape.

### 3.3. Model Performance Evaluation

**Figure 6:**
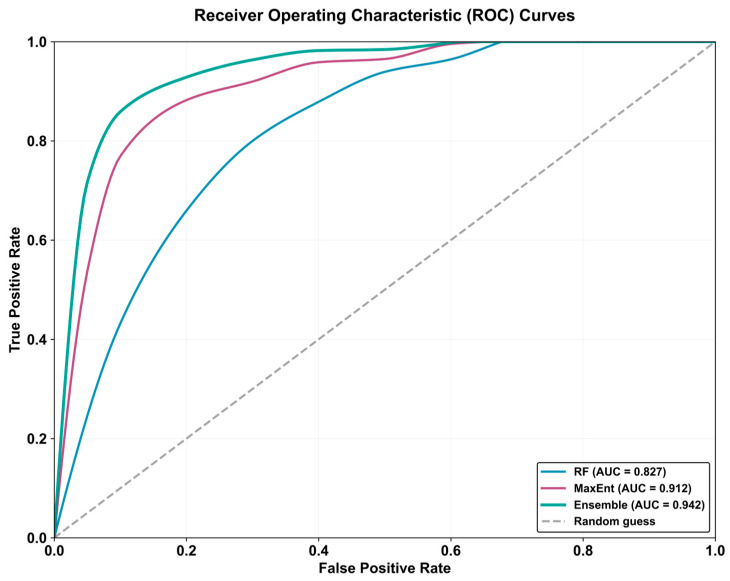
Performance Comparison of MaxEnt, RF, and Ensemble Approaches.

Receiver Operating Characteristic (ROC) curve analysis revealed distinct predictive abilities among the three modeling approaches (Figure 6). The Ensemble model showed the highest predictive performance (AUC = 0.942), indicating excellent habitat discrimination. MaxEnt also performed well (AUC = 0.912), while Random Forest demonstrated good predictive ability (AUC = 0.827). The stepwise increase in AUC from RF to MaxEnt to the Ensemble model highlights the progressive improvement in predictive accuracy achieved by combining models to produce a superior consensus prediction. RF’s lower performance, while still dependable, likely reflects its different handling of environmental variables and greater sensitivity to parameter tuning. These results emphasize how the choice of algorithm significantly influences predictions, with the Ensemble approach proving most effective for this conservation application.

### 3.4. Habitat Suitability by the Ensemble, MaxEnt, and RF Approaches

**Figure 7:**
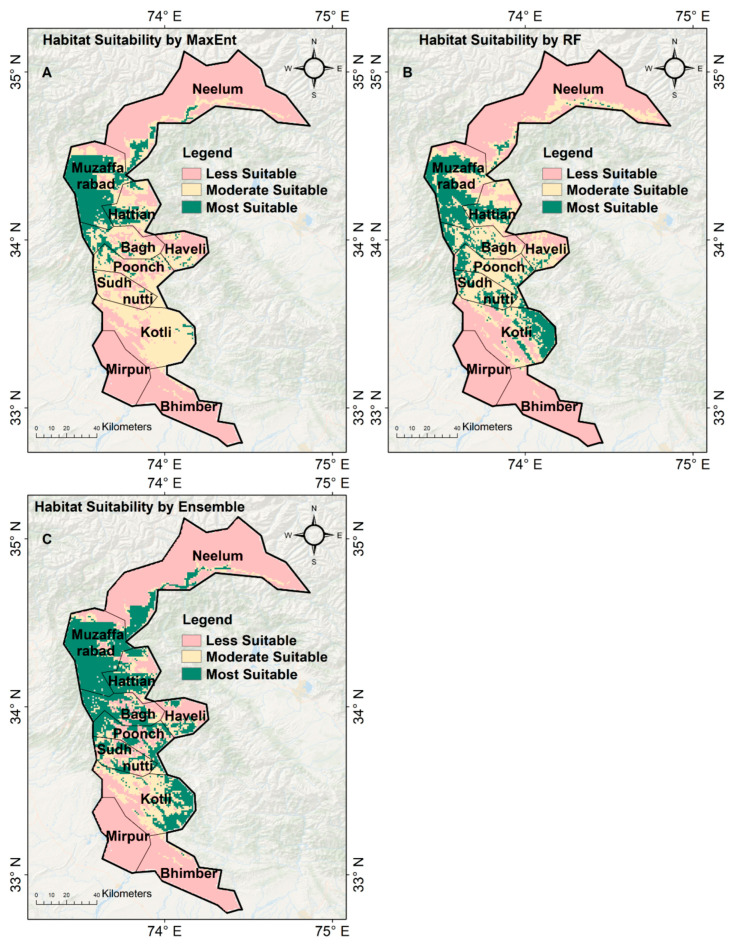
(**A**) Habitat suitability of the common leopard (*P. pardus*) by MaxEnt; (**B**) Habitat suitability of the common leopard (*P. pardus*) by RF approach; (**C**) Habitat suitability of the common leopard (*P. pardus*) by Ensemble.

**Figure 8:**
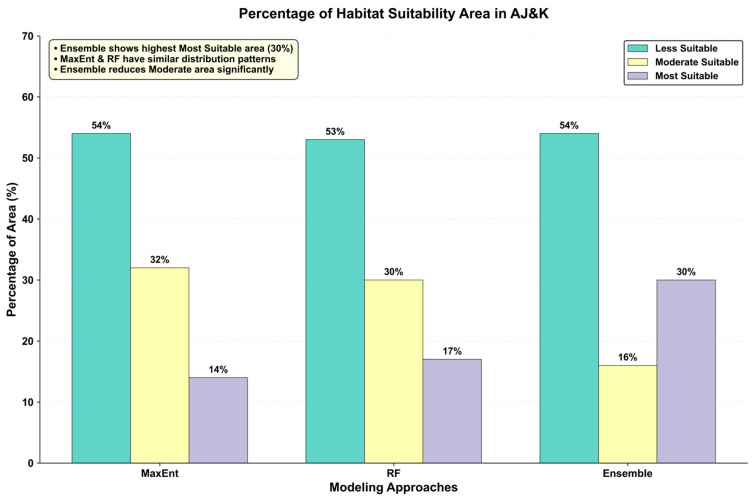
Percentage of habitat suitability.

The ensemble approach, which combines the outputs of both the MaxEnt and Random Forest (RF) models, provided the most refined and ecologically accurate prediction of habitat suitability for the common leopard (*P. pardus*) in Azad Jammu and Kashmir (AJ and K) (Figure 7C). This model classified 54% of the study area as less suitable, 16% as moderately suitable, and 30% as most suitable, providing a balanced view of leopard habitat suitability across the region (Figure 8).

Although all models were classified into the same suitability categories (as described in Section 2.5), the ensemble model predicted a higher proportion of “most suitable” habitat (30%) compared to MaxEnt (14%) and RF (17%). This difference reflects the integration of complementary model characteristics rather than a change in classification criteria. MaxEnt provides more conservative predictions focused on core suitable habitats, whereas RF captures additional suitable areas by modeling complex, non-linear relationships among environmental variables. The weighted ensemble combines these strengths, resulting in an expanded yet ecologically plausible delineation of highly suitable habitat.

The most suitable areas (30% of the study area) were mainly located in the northern and central regions, including Muzaffarabad, Neelum, Hattian, Poonch, and Sudhnutti. These regions are characterized by steep terrain, dense forests, and low human disturbance, making them ideal for leopards. High-elevation zones in these areas offer ample cover and prey, which are essential for leopard survival. The northern regions, especially Neelum and Muzaffarabad, feature rugged terrain and low human population density, along with habitat conditions likely to support prey species—both of which are crucial to leopards’ natural behavior. Additionally, these areas are less fragmented, allowing for continuous movement corridors vital for dispersal and breeding. In the central regions, such as Sudhnutti and Kotli, areas were also classified as most suitable, despite being more fragmented than the northern districts. These regions still retain significant forest cover and elevated terrain that support leopard populations; however, moderate human presence introduces risks of human–wildlife conflict, especially where human activities overlap with leopard territories.

The MaxEnt model (Figure 7A) showed a clear north-to-south gradient in habitat suitability, classifying 54% of the area as less suitable, 32% as moderately suitable, and 14% as most suitable (Figure 8). MaxEnt identified northern regions, such as Muzaffarabad, Hattian, Neelum, and Poonch, as the most suitable due to their rugged terrain, dense forests, and minimal human disturbance. However, in the central regions, such as Sudhnutti and Kotli, MaxEnt classified the areas as moderately suitable, reflecting the fragmented landscape of forested and farmed areas. While MaxEnt accurately captured the high suitability in the northern districts, its classification of the central region was less precise due to the complex mix of land uses.

**Figure 9:**
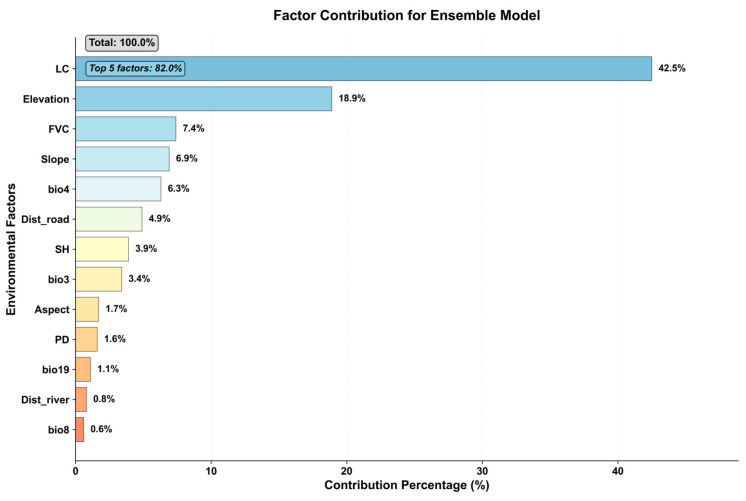
Factor contribution and response.

The Random Forest (RF) model (Figure 7B) produced a more varied suitability map than MaxEnt, classifying 53% of the study area as less suitable, 30% as moderately suitable, and 17% as most suitable (Figure 9). RF’s varied pattern provided a more detailed view of habitat suitability, capturing complex relationships among environmental factors such as land cover, elevation, and vegetation. The most suitable areas identified by RF were distributed across Muzaffarabad, Hattian, Bagh, Poonch, Kotli, and Sudhnutti, with suitable habitats in both northern and central regions. RF was especially good at identifying marginally suitable habitats in the central areas, such as Sudhnutti and Kotli, by analyzing complex interactions among elevation, fractional vegetation cover, slope, and land cover. While agricultural lands in the central regions were classified as moderately suitable, the model highlighted areas with dense forests and high elevations as highly suitable, aligning with the leopard’s habitat needs.

### 3.5. Variable Importance and Ecological Response

The ensemble model analysis provided important insights into the relative influence of environmental variables on habitat suitability for the common leopard (*P. pardus*) in Azad Jammu and Kashmir (AJ&K) (Figure 9). Land Cover (LC) emerged as the most influential variable, contributing 42.5% to the model predictions. A more detailed interpretation of land-cover classes indicates that forested areas—particularly dense and mixed forests—represent the most suitable habitats, as they provide cover, refuge, and favorable conditions for prey species. In contrast, agricultural lands, built-up areas, and sparsely vegetated or barren surfaces were associated with lower habitat suitability due to greater human disturbance and reduced ecological resources.

Elevation was the second most important factor, contributing 18.9%, highlighting the significance of higher-altitude zones that offer reduced human pressure and more intact natural habitats. These areas are often characterized by continuous forest cover and rugged terrain, which are conducive to leopard movement and shelter.

Other key contributing variables included Fractional Vegetation Cover (FVC) at 7.4%, reflecting the importance of vegetation density in providing concealment and supporting prey species, and slope at 6.9%, indicating a preference for rugged and steep landscapes that limit human access and provide natural protection. Bio4 (temperature seasonality), contributing 6.3%, suggests that leopards favor areas with moderate climatic variability that support stable habitat conditions.

Additional variables such as distance to roads (Dist_road), specific humidity (SH), and bio3 (isothermality) contributed 4.9%, 3.9%, and 3.4%, respectively, underscoring the combined influence of anthropogenic pressures and climatic factors. Lower contributions from variables such as aspect, population density (PD), and bio19 (precipitation seasonality), ranging from 1.7% to 1.1%, indicate a comparatively smaller role in determining habitat suitability.

Overall, these results demonstrate that land-cover composition, particularly the presence of forested habitats, along with elevation and vegetation structure, are the primary determinants of leopard habitat suitability in the study area. Human-related factors, including proximity to roads and population density, play a secondary but important role in shaping habitat use and potential movement patterns.

## 4. Discussion

The common leopard (*P. pardus*) is a highly adaptable species that thrives in a variety of environments. However, the availability and quality of suitable habitats are crucial for its survival, especially in regions with significant human presence. This study aimed to assess the habitat suitability of the common leopard in Azad Jammu and Kashmir (AJ and K) using an integrated ensemble modeling approach that combines MaxEnt and Random Forest (RF). The findings emphasize the complex interactions between ecological and human factors that shape leopard habitats and demonstrate how ensemble modeling can improve the accuracy of habitat predictions in fragmented and diverse landscapes.

In this study, we selected 28 environmental predictors based on known links to leopard habitat needs, including climate variables, vegetation and land cover, topography, and human-related factors. These factors are crucial in shaping leopard distributions, with climate affecting vegetation and prey availability, and topography influencing shelter and movement pathways. A thorough multicollinearity assessment using Pearson’s and Spearman’s rank correlation, along with Variance Inflation Factor (VIF) and Tolerance (TOL), confirmed that the predictors are independent and ecologically relevant. This detailed approach goes beyond simple collinearity tests, ensuring stable, easy-to-interpret results and thereby increasing the reliability of the modeling process, setting this study apart from others that rely on a single collinearity test [42,65].

In addition, global spatial autocorrelation analysis revealed significant clustering of common leopard occurrence points, confirming non-random spatial structure in the response variable and supporting the application of ensemble modeling to capture spatially structured habitat patterns.

The ensemble model, which combines the outputs of MaxEnt and RF, produced the most refined and ecologically consistent predictions of habitat suitability. This model classified 30% of the study area as highly suitable for leopards, with the northern and central regions, including Muzaffarabad, Neelum, Hattian, Poonch, and Sudhnutti, identified as key areas of high suitability. These regions are characterized by rugged terrain, high elevations, dense forests, and minimal human disturbance, making them ideal habitats for leopards. The MaxEnt model successfully identified the northern regions as highly suitable for leopards due to their rugged, forested landscapes. However, it struggled to capture the complexity of the central areas, which are more fragmented due to agricultural expansion and human encroachment. Conversely, the RF model produced a more dispersed suitability map, identifying suitable habitats across the northern and central regions and capturing complex nonlinear relationships among elevation, vegetation, and human activities. However, the RF model showed some tendency toward overfitting, especially in fragmented areas, suggesting that parameter tuning could improve its generalization.

Key ecological factors affecting habitat suitability for the common leopard in Azad Jammu and Kashmir (AJ and K) include land cover, elevation, fractional vegetation cover (FVC), slope, and temperature seasonality (Bio4). These factors are vital for determining the availability of resources such as cover, prey, and territorial space, which are essential to leopard survival [19,69]. Among these, land cover was the most influential, with dense forests and rugged terrain providing shelter, hunting grounds, and protection from human encroachment. Elevation and slope further enhance habitat suitability by providing high refuges and natural barriers to human activities, thereby shielding leopards from disturbance. Additionally, fractional vegetation cover (FVC) is crucial because it provides cover and prey, both of which are critical for successful foraging. Favorable seasonal temperature patterns (Bio4) ensure that climatic conditions remain suitable for leopard survival, maintaining a stable environment throughout the year. Although secondary, anthropogenic factors such as distance to roads and human population density also significantly influence habitat suitability, especially in fragmented areas. These factors contribute to human–wildlife conflict, particularly when leopards are pushed into marginal habitats by habitat loss. Proximity to human settlements and infrastructure increases the risk of negative interactions, underscoring the importance of accounting for human influence in conservation planning. Despite their secondary role, anthropogenic factors emphasize the need for conservation efforts to protect natural habitats and address challenges posed by human expansion.

While the ensemble approach provided a robust, ecologically consistent prediction of habitat suitability for the common leopard, several limitations remain. One major limitation is the lack of prey density data, which could have provided a more complete understanding of the factors affecting leopard habitat suitability. Prey availability is a vital part of carnivore habitats, and its absence in the models may have led to an underestimation of the importance of prey resources in shaping leopard distributions. Including prey density data in future models would improve the accuracy of habitat suitability predictions, ensuring that both predator and prey dynamics are considered in habitat assessments. Additionally, future studies could benefit from multi-temporal data to better capture seasonal changes in habitat use. The seasonality of leopard habitats is crucial, as leopards may use different areas depending on resource availability and climate conditions throughout the year. Understanding these seasonal patterns could provide a more complete view of habitat preferences and help develop adaptive conservation strategies that account for changing seasonal needs. Another important factor is the impact of human activities on leopard habitats. While anthropogenic factors were considered in this study, future research should focus more explicitly on strategies to reduce human–wildlife conflict. As human populations grow in the central regions of AJ and K, the overlap between leopard habitats and human activities is likely to increase. This could lead to more conflict events, such as livestock depredation, as leopards are pushed into marginal habitats due to habitat loss and fragmentation. Future efforts should prioritize creating wildlife corridors that connect fragmented landscapes and facilitate leopard movement. These corridors will be vital for maintaining genetic diversity, enabling safe dispersal, and reducing human–wildlife conflict, all of which are crucial to the species’ long-term survival.

## 5. Conclusions

This study provides a comprehensive assessment of habitat suitability for the common leopard (*P. pardus*) in Azad Jammu and Kashmir (AJ&K), using an ensemble modeling framework based on multi-source remote sensing data. The ensemble approach demonstrated high predictive accuracy and produced ecologically consistent, robust habitat suitability predictions. Approximately 30% of the study area was identified as highly suitable leopard habitat, primarily concentrated in the northern and central regions, including Muzaffarabad, Neelum, Hattian, Poonch, and Sudhnutti. These regions, characterized by rugged terrain, higher elevations, dense forest cover, and relatively low human disturbance, are critical for effective wildlife conservation efforts.

The analysis highlighted land cover, elevation, fractional vegetation cover, slope, and temperature seasonality as the dominant environmental drivers of habitat suitability, which are crucial for understanding the spatial dynamics of leopard habitats. These factors reflect the species’ reliance on structurally complex landscapes that provide cover, prey availability, and refuge from human disturbances. Anthropogenic factors such as proximity to roads and population density were also found to exert a secondary influence, especially in fragmented landscapes where human–wildlife interactions are frequent.

Despite the model’s strong performance, some limitations remain, including the absence of prey availability data and reliance on single-period environmental inputs. Future research incorporating prey density data and multi-temporal remote sensing datasets could improve understanding of seasonal habitat dynamics and further enhance habitat suitability models. From a conservation perspective, the identified high-suitability areas underscore priority zones for habitat protection, ecological corridor establishment, and human–wildlife conflict mitigation strategies, all essential to ensuring the long-term persistence of common leopard populations in this mountainous region.

## Figures and Tables

**Table 1:** Environmental predictors included in the suitability modeling process.

Category	Variable and Description	Abbreviation	Unit	Data Source
Climatic	Mean Annual Temperature	Bio1	°C	Worldclim
	Mean Diurnal Range (i.e., mean of monthly (max. temp.–min. temp.))	Bio2	°C	Worldclim
	Mean Annual Temperature Range (i.e., bio2/bio7 × 100)	Bio3	°C	Worldclim
	Temperature Seasonality	Bio4	°C	Worldclim
	Max. Temperature of Warmest Month	Bio5	°C	Worldclim
	Min Temperature of Coldest Month	Bio6	°C	Worldclim
	Annual Temperature Range (i.e., bio5–bio6)	Bio7	°C	Worldclim
	Mean Temperature of Wettest Quarter	Bio8	°C	Worldclim
	Mean Temperature of Driest Quarter	Bio9	°C	Worldclim
	Mean Temperature of Warmest Quarter	Bio10	°C	Worldclim
	Mean Temperature of Coldest Quarter	Bio11	°C	Worldclim
	Annual Precipitation	Bio12	mm	Worldclim
	Precipitation Level in Wettest Month	Bio13	mm	Worldclim
	Precipitation Level in Driest Month	Bio14	mm	Worldclim
	Precipitation Seasonality (i.e., coefficient of variation)	Bio15	%	Worldclim
	Precipitation Level in Wettest Quarter	Bio16	mm	Worldclim
	Precipitation Level in Driest Quarter	Bio17	mm	Worldclim
	Precipitation Level in Warmest Quarter	Bio18	mm	Worldclim
	Precipitation Level Coldest Quarter	Bio19	mm	Worldclim
	Specific humidity	SH	g/kg	FLDAS
Vegetation Cover	Fractional Vegetation Coverage	FVC	%	MOD13A2
Landscape	Land cover	LC	categorical	MCD12Q1.061
	Elevation	Elevation	m	GDEM V2/3
Topographic	Slope	Slope	°	Calculation in ArcGIS
	Aspect	Aspect	°	Calculation in ArcGIS
	Distance to road	Dist_road	Km	The Humanitarian Data Exchange
Human influenced	Population Density	PD	km	The Humanitarian Data Exchange
	Distance to the river	Dist_river	m	HYDROSHEDS

## Data Availability

Climatic variables included 19 bioclimatic parameters (Bio1–Bio19), sourced from WorldClim 2.1 (https://www.worldclim.org, accessed on 26 April 2025) at 1 km resolution. Additionally, specific humidity data were obtained from the Famine Early Warning Systems Network (FEWS NET) Land Data Assimilation System (FLDAS) dataset, accessed via Google Earth Engine (28 April 2025) at an 11,132 km resolution. Vegetation and land cover data were sourced from MODIS products. Fractional Vegetation Coverage (FVC) was calculated using the Normalized Difference Vegetation Index (NDVI) from the MOD13A2.061 product (1 km resolution, Google Earth Engine, accessed on 7 May 2025). The land cover classification was derived from the MCD12Q1.061 dataset (500 m resolution, Google Earth Engine, accessed on 23 March 2025). Topographic variables, including elevation (Digital Elevation Model, DEM), were obtained from the GDEM V2 and V3 datasets (Geospatial Data Cloud: https://www.gscloud.cn, accessed on 22 April 2025), which provide higher accuracy than previous versions. Using ArcGIS spatial analyst tools, we processed the DEM to generate slope and aspect layers. Anthropogenic factors, including distance to roads and population density data, were sourced from The Humanitarian Data Exchange (https://data.humdata.org, accessed on 11 May 2025) at 1 km resolution. Hydrological factor, distance to rivers, was extracted from the HYDROSHEDS database (https://www.hydrosheds.org, accessed on 11 March 2025) at an original resolution of 500 m.

## Supplementary Materials

The following supporting information can be downloaded at: https://www.mdpi.com/article/10.3390/s26103088/s1, Figure S1: Global Spatial Autocorrelation of Leopard Occurrence.
