# Supplementary material for: Multi-Source Remote Sensing and Ensemble Learning for Habitat Suitability Mapping of the Common Leopard (Panthera pardus) in Azad Jammu and Kashmir, Pakistan
Source: Sensors (Basel). 2026 May 13;26(10):3088. doi: 10.3390/s26103088 (PMC13210786; doi:10.3390/s26103088)
Supplement: Supplementary file 1 [file sensors-26-03088-s001.zip › sensors-4221844-Supplementary.pdf]

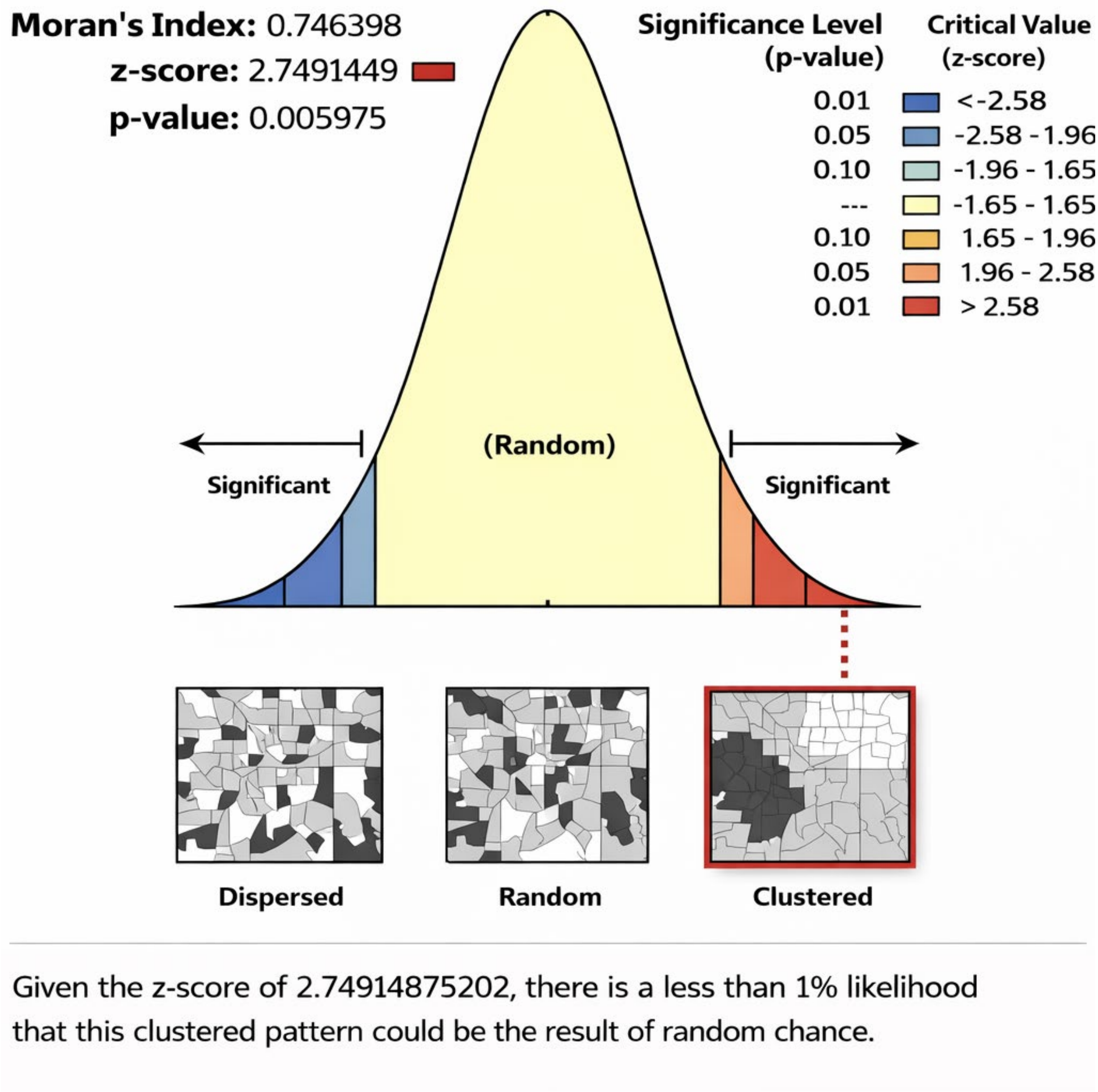

Figure S1: Global Spatial Autocorrelation of Leopard Occurrence
